# Supplementary material for: Increased EGFRvIII Epitope Accessibility after Tyrosine Kinase Inhibitor Treatment of Glioblastoma Cells Creates More Opportunities for Immunotherapy
Source: Int J Mol Sci. 2023 Feb 22;24(5):4350. doi: 10.3390/ijms24054350 (PMC10001577; doi:10.3390/ijms24054350)
Supplement: Supplementary file 1 [file ijms-24-04350-s001.zip › ijms-2058443-supplementary.pdf]

Supplementary Materials

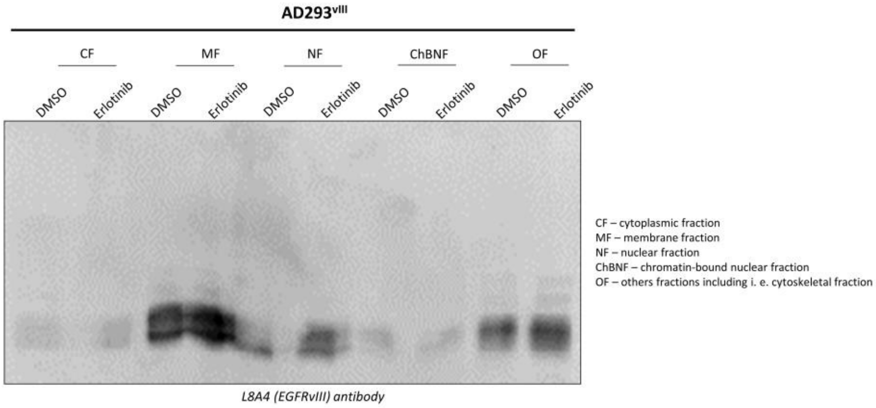

Supplementary Figure S1. Characteristics of EGFRvIII protein distribution into AD293^{vIII} cells after treatment with particular TKI.

(A) Western blotting analysis of EGFRvIII protein distribution in cell fractions after 1 h treatment with DMSO and erlotinib (10 µM) performed on AD293^{vIII} cell line. It was indicated that EGFRvIII occurred mainly in the cell membrane. Moreover, treatment with erlotinib increased protein amount in that compartment as well as in nuclear and cytoskeletal fractions. Analysis was conducted with the L8A4 antibody. CF—cytoplasmic fraction, MF—membrane fraction, NF—nuclear fraction, ChBNF—chromatin-bound nuclear fraction, OF—other fractions, e.g., cytoskeletal fraction;

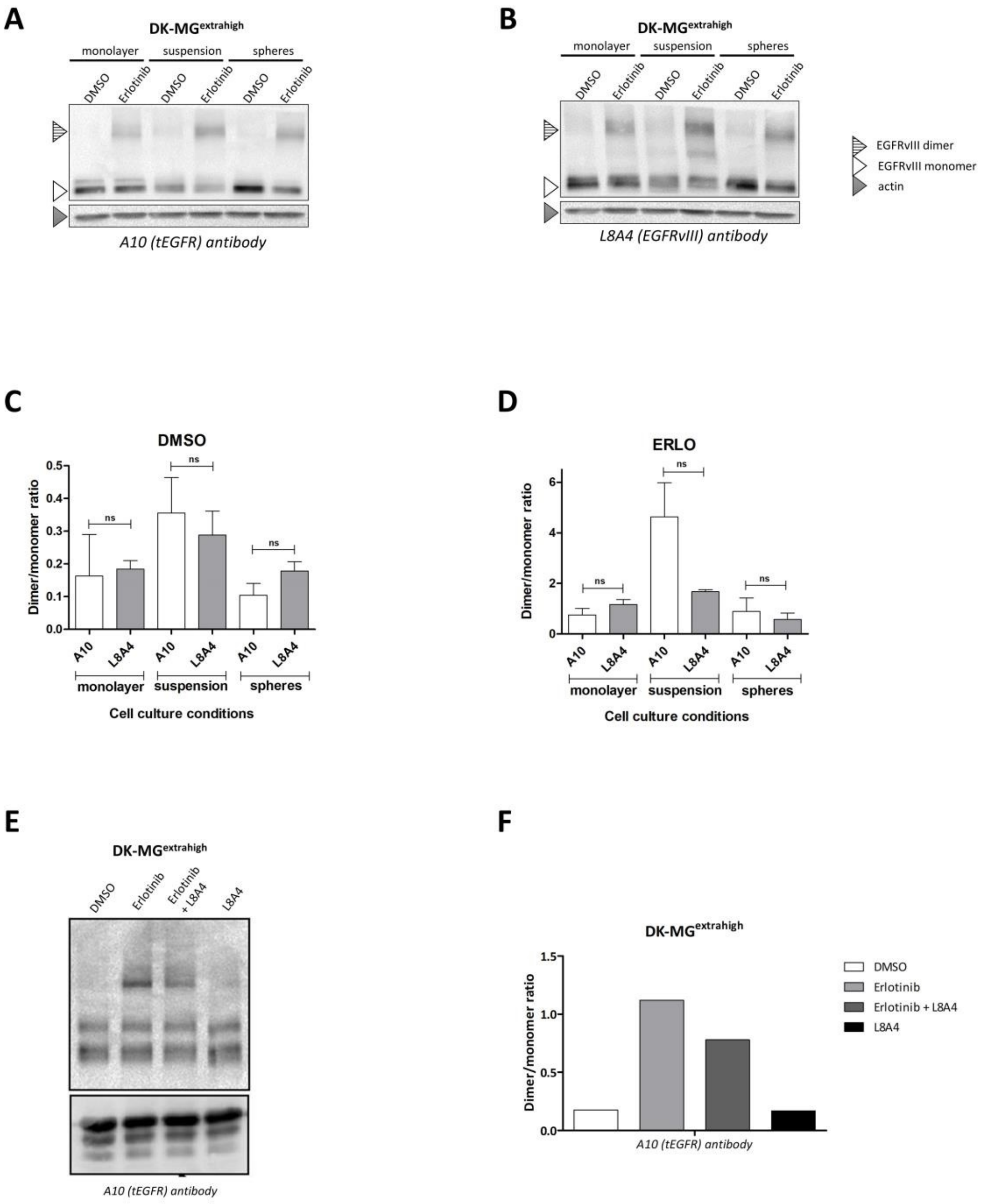

Supplementary Figure S2. Insight into antibody activity in various conditions.

(A,B) Influence of different types of cell culture—monolayer, suspension, and spheres—on the dimerization process in the case of DK-MG^{extrahigh}. Dimerization occurred in all examined conditions; however, the suspension method seemed to be most favorable for this process. Western blotting with either A10 (A) or L8A4 antibody (B).

(C,D) Statistical analysis of the ability of the L8A4 and A10 antibodies to recognize dimer formation in particular cell culture conditions (monolayer, spheres, suspension) and after 1 h treatment with DMSO (C) and erlotinib (D) indicated that there were no statistically significant differences between antibodies.

(E,F) In the case of DK-MG^{extrahigh}, the addition of the L8A4 antibody (10 μg/mL) to the cell culture blocked the EGFRvIII dimerization process, which was confirmed by semi-native Western blot (E) and densitometric analysis (F).

*Densitometric analysis (C,D) was obtained from three independent experiments, calculated by ImageJ, and data were analyzed with a two-tailed paired t-test, ns $p > 0.05$. WB from (E) was calculated by ImageJ, and data were analyzed with a two-tailed paired t-test, * $p \leq 0.05$.*

A

Primer	Sequence
EGFR-GW-F	GGGGACAAGTTTGTACAAAAAAGCAGCGTATGCGACCCTCCGGGACGGCC
EGFR-GW-R	GGGGACCACTTTGTACAAGAAAGCTGGGTTGCTCCAATAAATTCACTGC
C16S-F	CCTGTGGGGCCGACAGCTATGA
C16S-R	CTCGGACGCTCGAGCCGTGAT
C20S-F	AGCCAGTGGGGCCGACAGCTAT
C20S-R	CGGACGCACGAGCCGTGATC
C35S-F	AGGGCCTTGCCGCAAAGT
C35S-R	TCGCACTTCTTACTCTTGCGGA
C38S-F	CTTGCCGCAAAGTGTGTA
C38S-R	GCCCTTCGGACTTCTTACAC
C42S-F	AGGCCCTTCGCACTTCTTAC
C42S-R	AGGCCCTTCGCTCTTCTTAC
K721A-F	CGTCGCTATCGCCGAATTAAGAGAAGC
K721A-R	GGAATTTTAACTTTCTCACCTTCTGGG

B

Antibody	Host	Manufacturer	Dilution
total EGFR A-10	Mouse	Santa Cruz Biotechnology (sc-373746, USA)	1:500
total EGFR 1005	Rabbit	Santa Cruz Biotechnology (sc-03, USA)	1:500
phospho EGFR Y1068 D7A5	Rabbit	Cell Signaling (3777S, USA)	1:500
EGFRvIII L8A4	Mouse	Kerafast (EDK002, USA)	1:500
anti-Actin Clone C4	Mouse	Millipore (mab1501, USA)	1:4000
goat anti-rabbit IgG-HRP	Goat	Santa Cruz Biotechnology (sc-2004, USA)	1:4000
goat anti-mouse IgG-HRP	Goat	Santa Cruz Biotechnology (sc-2005, USA)	1:4000

C

Antibody	Host	Manufacturer	Dilution
total EGFR A-10	Mouse	Santa Cruz Biotechnology (sc-373746, USA)	1:300
EGFRvIII L8A4	Mouse	Kerafast (EDK002, USA)	1:300
EGFRvIII L8A4	Rabbit	Absolute Antibody (Ab00184-23.0, USA)	1:300
anti-mouse AlexaFluor594	Donkey	Life Technologies (USA)	1:500
anti-rabbit AlexaFluor488	Donkey	Life Technologies (USA)	1:500

Supplementary Table S1.
(A) Primer sequences. (B) Antibodies used in Western blot analyses. (C) Antibodies used in immunofluorescence analyses.

A

	C16	C20	C35	C38	C42
C16	X	11.33	12.09	21.01	21.18
C20	11.33	X	Bridge	9.82	9.88
C35	12.09	Bridge	X	9	9.46
C38	21.01	9.82	9	X	Bridge
C42	21.18	9.88	9,46	Bridge	X

B

Free C	diAnna	DICON	SCRATCH	EDBCP	DISULFIND	SYSCON	Score
C16	1	0	1	0	0	1	3
C20	0	0	0	0	0	0	0
C35	0	1	0	0	0	0	1
C38	0	1	1	0	0	0	2
C42	0	0	1	0	0	0	1

Supplementary Table S2.

(A) Distances of SG atoms in individual cysteines of the EGFR domain III. (B) Prediction results based on sequence (1—slow, 0—bound);
